# A Welcome Fashion

**Published:** 1874-11

**Authors:** 


### A WELCOME FASHION.

WE have been pleased to discover a growing taste for flowers and shrubs all over the city. There is no surer evidence of an elevated taste than the cultivation of those

> Floral apostles, that in dewy splendor
> Weep without woe and blush without a crime, which a benignant Creator has lavished upon those who are disposed to cherish and care for them. With real satisfaction we have observed that the various hotels here have come to consider it advisable to adorn their windows with floral displays. The Fifth Avenue Hotel employs accomplished florists by the season to keep in perfect condition the fine flower-beds on the south side of the great edifice; and the admirable Hotel Brunswick is rendered very attractive in appearance by the enormous and elaborate vases of flowers in bloom which adorn the front of that establishment. The St. James, just re-opened in grand style, follows the popular taste, and blossoms like the rose in all directions. Our people are rapidly growing in their appreciation of the advantage of flowers as ornaments; and we trust the time is not far distant when every home in the city will be blest by their refining and elevating influence.

